# Comparison of Attenuated and Virulent Strains of African Swine Fever Virus Genotype I and Serogroup 2

**DOI:** 10.3390/v15061373

**Published:** 2023-06-14

**Authors:** Natalia Kholod, Andrey Koltsov, Sergey Krutko, Edan R. Tulman, Sanzhi Namsrayn, Gerald F. Kutish, Sergey Belov, Alexey Korotin, Mikhail Sukher, Galina Koltsova

**Affiliations:** 1 Laboratory of Viral Genomics, Federal Research Center for Virology and Microbiology, 601125 Pokrov, Russia; natkholod@yandex.ru (N.K.); kolcov.andrew@gmail.com (A.K.); sergejjkrutko@gmail.com (S.K.); namsrayn.szh@gmail.com (S.N.); belovsergej371@gmail.com (S.B.); alescha.korotin@yandex.ru (A.K.); suhermail@mail.ru (M.S.); 2 Department of Pathobiology and Veterinary Science, Center of Excellence for Vaccine Research, University of Connecticut, Storrs, CT 06269, USA; edan.tulman@uconn.edu (E.R.T.); gkutish@netzero.net (G.F.K.)

**Keywords:** ASFV, attenuated strain, complete genome sequencing

## Abstract

African swine fever (ASF) is a contagious disease of pigs caused by the ASF virus (ASFV). The main problem in the field of ASF control is the lack of vaccines. Attempts to obtain vaccines by attenuating the ASFV on cultured cell lines led to the production of attenuated viruses, some of which provided protection against infection with a homologous virus. Here we report on the biological and genomic features of the attenuated Congo-a (KK262) virus compared to its virulent homologue Congo-v (K49). Our results showed differences in in vivo replication and virulence of Congo-a. However, the attenuation of the K49 virus did not affect its ability to replicate in vitro in the primary culture of pig macrophages. Complete genome sequencing of the attenuated KK262 strain revealed an 8,8 kb deletion in the left variable region of the genome compared to the virulent homologue K49. This deletion concerned five genes of MGF360 and three genes of MGF505. In addition, three inserts in the B602L gene, genetic changes in intergenic regions and missense mutations in eight genes were detected. The data obtained contribute to a better understanding of ASFV attenuation and identification of potential virulence genes for further development of effective vaccines.

## 1. Introduction

African swine fever (ASF) is an acute viral hemorrhagic disease of domestic swine with a mortality rate close to 100% [1,2,3]. After being imported from Eastern Africa to Georgia [4], ASFV of genotype II has been circulating in Eastern Europe since 2007, in the European Union since 2014 and in Asia since 2018 (OIE). The spread of the disease outside Africa has become a global threat with enormous economic losses for swine-raising countries as well as ecological consequences [5].

ASFV is a large icosahedral cytoplasmic virus and the only member of the *Asfarviridae* family [6]. Based on the sequence of the B646L gene encoding the p72 capsid protein, 24 genotypes of the ASFV were identified [7,8]. Eight ASF virus serogroups have been identified, although most likely there are more of them [9,10,11]. Of particular importance for the development of a vaccine is that protective immunity against ASF seems to depend on the serotype, since viruses within the serogroup provide cross-protection of animals from each other [9,11,12,13,14]. In addition, HAI typing and classical vaccination/challenge experiments places the ASFV into discrete serogroups, which are not always separated by the usual typing of the P72 capsid protein gene [13]. We have previously demonstrated that the ASFV genetic locus encoding CD2v and C-type lectin proteins mediates the serological specificity of HAI and that CD2v/lectin genotyping provides a simple method of grouping ASFV by serotype [13].

Since ASF spreads quite quickly, and treatment is prohibited, the development of vaccines for the prevention of ASF infections is one of the priorities of veterinary medicine. To date, among the various approaches to the development of vaccines, the greatest success has been achieved using live attenuated strains of the ASF virus. In parallel with the search for naturally attenuated strains, scientists tried to obtain attenuated strains by passages of virulent viruses on various cell cultures. Early studies have shown that after continuous passage in cells, the virulence of the virus decreases, and inoculation of pigs with such a virus could protect against infection with a homologous virulent strain [11,12,15]. Meanwhile, other studies have demonstrated that some viral strains, although they showed reduced virulence, did not provide protection against homologous viruses [16]. In addition, the features of the same ASFV strains obtained as a result of their passage in different cell lines differed significantly [17]. Since the attenuated ASFV strains were obtained by sequential passaging, it was not initially known what changes were introduced into the viral genome as a result. Later, efforts were made to identify the genetic changes responsible for the loss of virulence. Thus, complete genome sequencing of the attenuated strains showed the presence of significant deletions in the left and right variable ends of their genome [16,18,19,20]. In addition to obtaining vaccine strains, attenuated viruses are of great importance for understanding the genetic changes that occur during adaptation to cells. The complete genome sequencing analysis helps to determine the function of viral genes in maintaining virulence and immunogenicity, which makes it possible to carry out targeted genome modifications to obtain recombinant live vaccines.

Here we present an analysis of the complete genome sequences of the attenuated Congo-a (KK262) in comparison with its virulent homologue Congo-v (K49). ASFV K49 is a highly virulent strain with a lethal dose of 100 HAU, and ASFV KK262 is an avirulent variant at a dose of 10^6^–10^7^ HAD50 [15]. Both strains belong to genotype I and serogroup 2 based on p72 and CD2v sequences, respectively. In addition, these strains were assigned to seroimmunotype 2 in accordance with the results of pigs’ vaccination/challenge experiments and serological tests (HAI) [10,11]. The attenuated strain KK262 was obtained by multiple passages in primary (porcine bone marrow cells) and continuous (SPEV) cell cultures and was adapted on a continuous COS-1 cell line [15]. In addition, _Ы_KK262 is able to grow on several continuous cell lines such as A4C2/9k, SK6, PTP, Vero, and MA-104, whereas the parental strain K49 replicates only in primary cell cultures (our unpublished data).

Previously, we showed that double immunization with a virulent strain of ASFV KK262 completely protects animals from death and the development of clinical signs after infection with a homologous strain of ASFV K49 [15,21]. Despite the fact that the virulence of both strains was described earlier, no detailed study of the attenuated strain was conducted. Thus, we have shown that the deletion of the CD2v gene in the KK262 strain does not affect the replication of the virus in vitro, but no comparison with its virulent homologue was conducted [21]. The aim of this study was to investigate in detail the replication of the attenuated strain KK262 in comparison with the parental virulent strain K49 both in vitro and in vivo. In addition, we conducted a comprehensive comparative analysis of genetic characteristics to identify possible molecular determinants of virulence and adaptation of the virus to replication in cell cultures.

## 2. Materials and Methods

### 2.1. Cell Cultures and Viruses

The attenuated ASFV Congo-a (strain KK262, Genotype I, Serogroup 2) and parental virulent ASFV Congo-v (strain K49, Genotype I, Serogroup 2) were received from the reference collection of the Federal Research Center for Virology and Microbiology in Russia. The strain K49 was initially isolated in 1949 from a domestic pig (*Sus scrofa domesticus*) in the Katanga Province of the Democratic Republic of the Congo [22]. The ASFV strain KK262 is a derivative of the high virulent strain K49 with 50 serial passages in porcine kidney cell lines (SPEV) and 262 passages in porcine bone marrow cell cultures [15].

Primary swine macrophage cultures were prepared from defibrinated blood using Lymphocyte separation media. Cells were cultured in 96-well plates (Corning, Corning, NY, USA) (1.3 × 10^6^/well) containing RPMI 1640 medium supplemented with 30% (*v*/*v*) plasma, 10% (*v*/*v*) fetal bovine serum (Gibco, Hong Kong) and antimycotic-antibiotic (Gibco) at 37 °C with 5% CO_2_ for 24 h. The adhering cells were rinsed with the same macrophage medium and used in assays after 48 h.

Virus titration was performed in 96-well plates by visualizing hemadsorption in primary swine macrophage cultures. Titers were expressed as mean hemadsorption doses (HAD50), according to the Reed–Muench method [23].

The growth curves of the attenuated ASFV Congo-a and the parental virulent ASFV Congo-v were measured in primary swine macrophage cultures using 48-well plates. The cultures were infected with the virus at MOI of 1 and 0.1. After 1 h of adsorption, the virus was removed; the cells were rinsed with PBS and incubated at 37 °C with 5% CO_2_ in macrophage medium. The cells were harvested at 0, 24, 48, 72, and 96 h post-infection (hpi) and frozen until lysates were used for determination of HAD50/mL titers in primary swine macrophage cell cultures.

### 2.2. Animal Experiment

Animal experiments were performed under biosafety level 3 (BSL-3) conditions at the Federal Research Center for Virology and Microbiology facility, following a protocol approved by the Institutional Animal Care and Use Committee (IACUC). Each group was housed in an isolated room of the FRCVM animal facility during the entire experiment (including an acclimatization period of one week prior to infection).

In vivo replication and virulence of attenuated ASFV Congo-a and parental virulent ASFV Congo-v were assessed using Large White pigs (male/female), aged 2–2.5 months (weighing 15–18 kg), commercial-breed swine. Piglets were diagnosed as free of specific pathogens (data not shown) and were not vaccinated against any infectious disease.

Pigs from group 1 (n = 10) were infected intramuscularly with 2 × 10^6^ HAD50 of Congo-a. Pigs from group 2 (n = 10) were infected intramuscularly with 10^3^ HAD50 of Congo-v. Clinical signs and rectal body temperature were recorded daily throughout the experiment. Clinical assessment of ASF was conducted in 4 different categories (behavior and mentation, neurologic signs, defecation, body temperature). The clinical signs were assigned numerical values based on severity and significance as described by Howey et al., 2013 [24]. The sum of points was recorded as a clinical score. Clinical scores were recorded daily for each pig.

Survival and time-to-death were recorded as previously described [14]. To assess viremia, blood samples (blood with EDTA) were taken from the jugular vein at 0, 7, 14, 21, and 28 days post-inoculation (dpi). During necropsies, five tissues (spleen, liver, lung, mesenteric lymph node, sub-mandibular lymph node) were collected from infected pigs and frozen at −70 °C.

Quantitative PCR of ASFV in blood and tissues samples (10% suspension) was performed as previously described [25]. DNA from blood and tissues samples was isolated using the ExtractDNA Blood (Evrogen, Moscow, Russia) according to the manufacturer’s instructions.

### 2.3. Next Generation Sequencing (NGS)

The total genomic DNA of the ASFV was extracted from the primary swine macrophages cells infected with Congo-a or Congo-v. Briefly, each virus was subjected to a single passage on primary swine macrophage cells in a T-25 flask, and cells were collected by centrifugation. Then, the cells were resuspended in cold sterile PBS (Gibco) and used to extract viral DNA using a DNeasy blood and tissue kit (Qiagen, Hilden, Germany) according to the manufacturer’s instructions. The quality and quantity were measured using a spectrophotometer NanoDrop (Thermo Scientific, Waltham, MA, USA). Genomic library was prepared using NEBNextNEBNext^®^ Ultra™II DNA Library Prep Kit for Illumina^®^ (NEB, Ipswich, MA, USA) and sequencing of 150 bp paired-end reads was performed on an HiSeq1500 (Illumina, San Diego, CA, USA). The final library quality and quantity were analyzed in Bioanalyzer 2100 (Agilent Technologies, Santa Clara, CA, USA) and Qubit 2.0 Fluorometer (Thermo Scientific, Waltham, MA, USA).

Gene locations were predicted by algorithms of GLIMMER3 v.0.2 [26]. Final protein-coding gene calls of K49 were made manually, based on the presence of valid start and stop codons. Annotation of valid ORFs was performed by comparing K49 nucleotide sequence to closely related ASFV genomes available in the NCBI database using BLAST (https://blast.ncbi.nlm.nih.gov/Blast.cgi, accessed on 19 April 2023). ASFV KK262 genome was annotated with the aid of Genome Annotation Transfer Utility software [27], using the parental K49 strain as a reference (GenBank accession numbers MZ202520.1). The complete genome sequences of the Congo-a (KK262) strain were deposited in GenBank with accession number OM249788.1.

The graphic representation of genomes and ORFs alignments were made using Mauve [28]. MEGA-X was used to generate sequence alignments using the ClustalW algorithm [29,30]. In order to find and analyze indels and SNPs, sequence alignments were analyzed base-by-base in Aliview [31].

## 3. Results

### 3.1. In Vivo Replication and Virulence in Susceptible Animals

In vivo replication and virulence of the attenuated ASFV Congo-a and the parental virulent ASFV Congo-v were assessed in an animal experiment using different doses (2 × 10^6^ HAD50 and 1 × 10^3^ HAD50) for these viruses, respectively.

**Figure 1:**
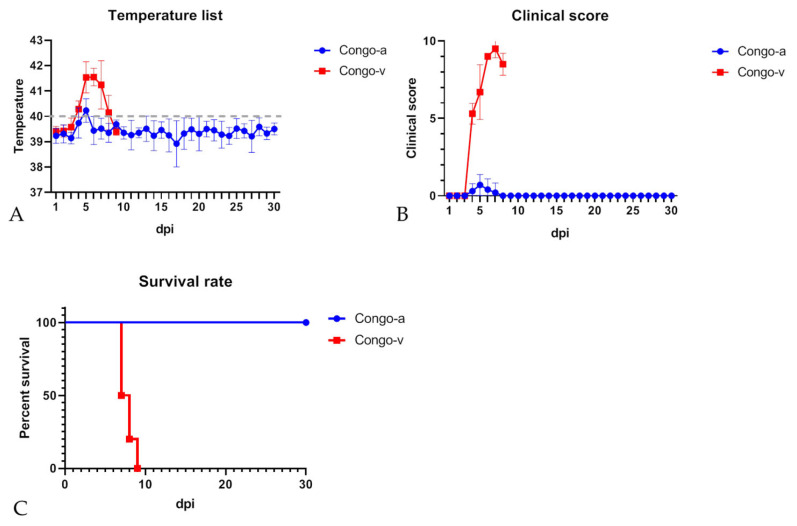
Body temperature (**A**), clinical signs (**B**) and lethality kinetics (**C**) in pigs inoculated with ASFV strains Congo-a (KK262) and Congo-v (K49). The dashed line represents the fever-cutoff (40 °C) (**A**). Pigs were inoculated with Congo-a (strain KK-262) (blue line) or Congo-v (strain K49) (red line). Data on body temperature and clinical score are shown as average values and their SD in each of the groups. The analysis was conducted using Graphpad Prism software version 8.0.1.

Transient fever was detected in all pigs immunized with Congo-a at 4–6 days dpi (Figure 1A and Figure S1A). All pigs from the immunized Congo-a group, regardless of their gender and body weight, did not show any clinical signs associated with the disease (except for the fever) until the end of the observation period (30 dpi) (Figure 1B and Figure S1B). All animals showed good health, appetite, and activity. After infection with ASFV Congo-v, animals from Group 2 developed typical clinical symptoms of ASF, including hyperthermia (from 3–5 dpi), depression (from 3–5 dpi), anorexia (from 4–5 dpi), and skin cyanosis (from 4–5 dpi) (Figure 1A,B). Nevertheless, there was no bleeding from the nose or rectum. Pigs in the Congo-v group were dead or found in a moribund state and euthanized at 7–9 dpc (Figure 1C). We did not indicate significant differences in the manifestation and duration of the disease or the spectrum of clinical signs in animals within each group.

**Figure 2:**
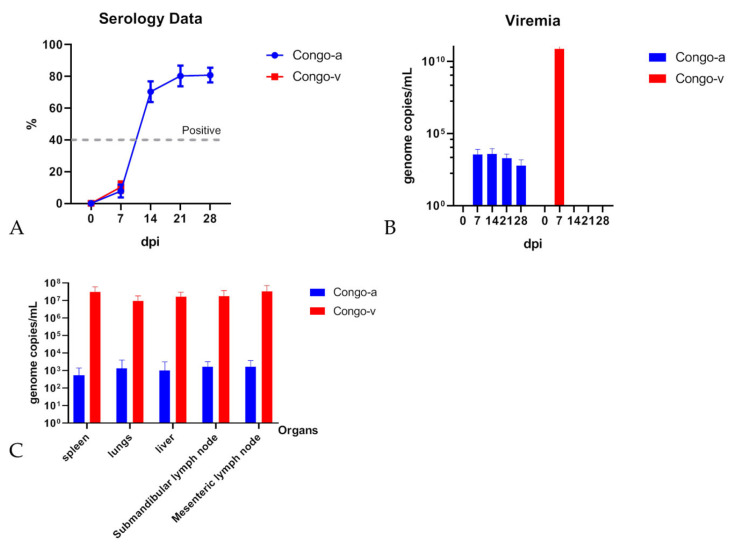
Antibody response to ASFV (**A**), kinetics of ASFV genomes in blood (**B**) and viral load in organs (**C**) detected in pigs IM inoculated with 10^6^ HAD50 ASFV Congo-a or 10^3^ HAD50 ASFV Congo-v. Antibody responses to ASFV (**A**) measured using ELISA in pigs immunized with ASFV Congo-a or Congo-v at 0–28 dpi. The dashed line indicates a threshold value of 40%. The detection of the ASFV genome was conducted in blood (**B**) and organs (**C**) by the qPCR. The results are presented as genome copies/mL. Data on antibody response to ASFV, the amount of ASFV genome in blood and organ samples are shown as average values and their SD in each of the groups. The analysis was conducted using Graphpad Prism software version 8.0.1.

The presence of anti-ASFV antibodies in the serum of animals inoculated with Congo-a and Congo-v was tested by ELISA. All animals inoculated with Congo-a were found to be positive (S/P > 40%) for anti-ASFV antibodies 14–42 days after inoculation (Figure 2A and Figure S2). All serum samples taken from animals infected with Congo-v were negative, which is associated with the early death of animals.

The ASFV genome (10^2^–10^4^ copies/mL) was detected by qPCR in blood samples collected from animals immunized with Congo-a at 7 to 30 dpi (Figure 2B and Figure S3A). In most animals, the largest number of copies of the viral genome was detected on 7–14 dpi (10^3^–10^4^ copies/mL), with the exception of two animals in whose blood the ASFV genome was not detected 7 days after infection with the attenuated Congo-a virus. Despite this, both of these animals had fever at 5–6 dpi. The ASFV genome was detected in the blood at 14 days (animal #1/10) and at 21 days (animal #1/3) post infection (Figure S3A). Interestingly, these animals showed the lowest maximum value of the viral genome in the blood (8 × 10^2^ copies/mL). Viraemic loads with a high amount of ASFV genome (10^3^–10^11^ copies/mL) were detected in animals infected with Congo-v at 3 dpi and persisted until death, reaching a maximum at 7 dpi (Figure 2B and Figure S3A). There were no significant differences in viral load and genome detection time in animals of this group.

It is important to note that only very low or undetectable amounts of the ASF virus genome (10^2^–10^3^ copies/mL) were detected in the organs of animals immunized with Congo-a (Figure 2C and Figure S3B). Moreover, in two animals inoculated with Congo-a (#1/5, #1/10), the genome was found only in the lymph nodes (Figure S3B).

ASFV DNA was detected by qPCR using primers specific to the B646L gene. Due to the low titer of the virus in animal organs, it was not possible to isolate and confirm the presence of a replication-competent virus. On the contrary, high titers of the ASF virus and ASFV genome (10^7^–10^8^ copies/mL) were found in organ samples of dead or euthanized animals infected with the Congo-v virus (Figure 2C and Figure S3B).

### 3.2. Replication of ASF Viruses in Primary Swine Macrophage Culture

Swine macrophages are the main cellular target when pigs are infected with ASFV, so it was critical to assess the replication of both viruses in swine macrophages. Congo-a and Congo-v ASF viruses have demonstrated the ability to grow efficiently in the primary swine macrophage culture with the formation of similar rosettes during hemadsorption (in the presence of erythrocytes).

In vitro virus replication was evaluated in swine macrophage culture using multistep growth curves. The cells were infected with both viruses at a MOI of 1 or 0.1. At 0, 24, 48, 72 and 96 hpi, the virus yield was quantified by HAD50/mL titers in primary swine macrophage cell cultures. The infection multiplicity of 0.1 was used to ensure multiple replication cycles during the experiment.

**Figure 3:**
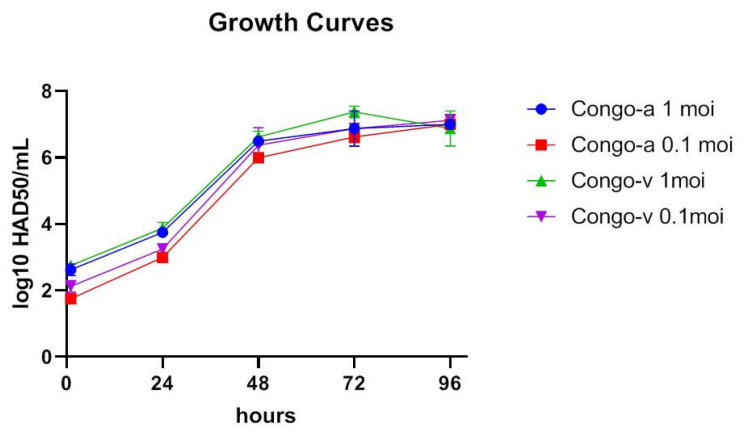
Kinetics of growth in vitro by Congo-v (K49) virus and attenuated Congo-a (KK262) virus in primary swine macrophage cultures: multistep growth curves in primary swine macrophage culture expressed in log10 HAD50/mL. The analysis was conducted using Graphpad Prism software version 8.0.1.

No significant differences in virus titers were observed until the end of the experimental period (96 hpi) (Figure 3). Thus, we have shown that differences in in vivo replication and virulence of Congo-a and Congo-v viruses are not related to in vitro replication of the virus in macrophages.

### 3.3. Comparison of Congo-a and Congo-v Genomes

The annotation of the complete genome of the Congo-v strain (K49) was published by us earlier [22]. The K49 genome sequence has a length of 189,523 bp and contains 189 open reading frames (ORFs). Now we present an annotation of the complete genome of the Congo-a strain (KK262). A comparison of the two sequenced genomes revealed that K49 and KK262 genomes are collinear and largely identical, but KK262 has 180,891 bp and contains only 181 ORFs. Table 1 shows all the detected differences in the genome of the attenuated strain KK262 compared to the parental virulent strain K49.

**Figure 4:**
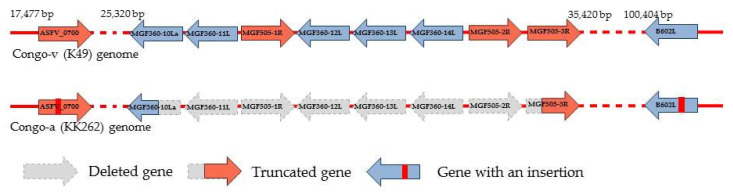
Deletion of the genes of the MGF360/MGF505 families and insertion in the B602L gene found in the KK262 genome compared to the K49 genome.

A comparison of the two genomes showed that the major changes were mapped to a region called the left variable region (LVR) (Figure 4, Table 1). The biggest difference in the KK262 genome is the deletion of 8811 nucleotides in the region of multigenic families (MGF360/MGF505). This deletion found in the attenuated virus is responsible for the differences in genome length observed between strains. The deletion affected five genes of the MGF360 (MGF360-10La, MGF360-11L, MGF360-12L, MGF360-13L and MGF360-14L) and three genes of the MGF505 (MGF505-1R, MGF505-2R and MGF505-3R) (Figure 4).

Two insertions were also found in two other LVR genes, MGF110-10L/11L and ORF ASFVK49_0700. The first insertion in KK262 is located at position 12363-12367 in the poly-C area and consists of five cytosine (C) bases. This can cause a frameshift and translation changes of the MGF110-10L/11L fusion protein. KK262 ORF0700, which encodes an uncharacterized protein, contains the insertion “TAGTTTAAACAT” repeated three times. Interestingly, the same nucleotide pattern is present in the original gene sequence, but only in two copies, while the KK262 version has a total of five.

In addition to coding regions, three deletions and one insertion were found in intergene regions (IGRs) of KK262 LVR. Deletion of 12 bases located between two ORFs of unknown function downstream of MGF360-4L (ASFVK49_0660/0665), deletion by 4 nucleotides located between ORFs ASFVK49_0700/0720, and insertion by 20 nucleotides in in Tandem Repeat Region located between ORF 00600 and A104R genes. The insertion is located between genes MGF360-9L and MGF360-10Lb and contains a single repeat of “TAACCATGTTA” making a total of three copies of the repeat in KK262 and two in parental K49. Another insertion was found in the IGR of the central region of the ASF virus. It is located between C315R and C147L genes, where an extra copy of the “TTTAAACTAAACG” repeat is added to the KK262 sequence.

**Figure 5:**
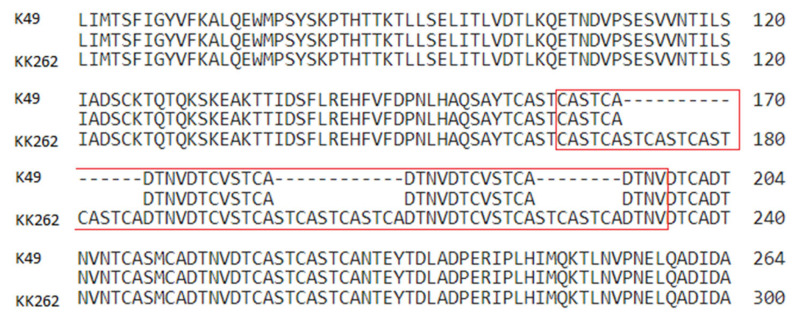
Pairwise alignment of the B602L gene on K49 (Congo-v) with KK262 (Congo-a), insertions are located in a red box.

In addition, the genome of the attenuated strain has multiple insertions in the central hypervariable region (CVR) of the B602L gene (Figure 5, Table 1). These inserts of tandem repeats resulted in an elongation of the central part of the B602L protein by 36 amino acids.

There are a number of single-nucleotide mutations obtained by the KK262 strain during passage in cell culture. Most of them are located in genes that are well conserved across ASFV species. One substitution was found in the genes CP530R (encoding pp62 polyprotein), NP1450L (encoding RNA polymerase subunit 1), E199L (encoding membrane fusion protein pE199L) and I8L (encoding a non-essential early protein) (Table 1). Several genes contain more than one amino acid substitution. The D250R gene coding for mRNA decoupling enzyme has three substitutions, one of which is located at the N-terminus of the protein, and two of them are close to the C-terminus. However, all three mutations do not belong to the catalytic domain of the protein. In addition, the E120R gene encoding the structural protein p14.5 has two missense mutations at the C-terminus (Table 1).

## 4. Discussion

It has been shown that live attenuated strains of the ASF virus obtained by adaptation to cell culture, as well as naturally attenuated field isolates, induce different degrees of protection of pigs from infection with homologous virulent isolates. Many studies have confirmed that virulent field isolates after sequential passage in cell lines demonstrate reduced virulence for pigs [18,32,33]. We have demonstrated that the parental Congo-v (K49) and the attenuated Congo-a (KK262) viruses differ significantly in their virulence. In addition, higher virus titers in the organs and blood of animals infected with the Congo-v strain indicate a significant difference in the replication of attenuated and virulent variants of the virus. We have previously shown that the ASF Congo-a virus causes strong protective immunity in susceptible animals when they are sequentially infected with the virulent Congo-v virus [15]. Thus, the genetic changes obtained by the Congo-a virus as a result of multiple passaging are associated only with the replication of the virus in vivo and its virulence and did not affect the genes associated with the induction of a protective immune response. In addition, our results showed that differences in in vivo replication and virulence of the Congo-a virus are not associated with an impairment of its replication on the primary macrophages in vitro. Thus, the attenuation of the K49 virus did not affect its ability to propagate effectively in vitro in the primary culture of pig macrophages.

The process of adaptation of the ASFV to reproduction in cell lines is often accompanied by significant modifications of the viral genome [18,33,34,35,36]. These genomic changes usually affect variable regions, especially the ends of the genome. Complete genome sequencing of the attenuated BA71V strain revealed that large gene deletions occurred between MGF360-9L to MGF505-7R and I7L to MGF360-18R on the left (LVR) and right (RVR) variable regions, respectively [18]. Later, another study showed the presence of three large deletions in the left and one in the right variable regions of the same strain BA71V [19]. Deletion of genes of multigenic families in the LVR of the genome was found in many attenuated strains of the ASFV (OURT88/3 [36], Pr4Δ35 [37], E70ΔNL [38] and ASFV-G/VP110 [16]). However, these deletions often lead not only to a weakening of virulence, but also affect the ability of the ASFV to grow in macrophage cell cultures. Recently, similar results were obtained after 121 passages of the virulent ASFV in HEK293T cells [20]. The total deleted gene sequence in ASFVP121 was about 25 kb long and included 22 MGF genes in the LVR of the genome. The adapted virus replicated well in HEK293T cells, as well as in Vero cells, but demonstrated a reduced ability to replicate in the culture of peripheral alveolar macrophages [20]. In addition to in vitro experiments, it was shown that the recombinant virus (ASFV-G-ΔMGF) with deletion of six genes belonging to MGF360 and MGF505 (MGF505-1R, MGF360-12L, MGF360-13L, MGF360-14L, MGF505-2R, and MGF505-3R)—constructed from a highly virulent ASFV isolate Georgia 2007 (ASFV-G)—was completely attenuated in pigs and provided reliable protection against virulent ASFV-G [39,40].

Complete genome sequencing of the attenuated KK262 strain also revealed an 8.8 kb deletion in the LVR of the genome compared to virulent homologue K49 strain. This deletion concerned five genes of the MGF360 and three genes of MGF505, which suggests that these changes are sufficient to lose virulence, but at the same time the ability of the virus to replicate in macrophage culture in vitro remains unchanged. Despite the fact that the functions of many MGF genes are not fully understood, there is some evidence of their role in the virulence of the ASF virus. For example, it has been shown that a single deletion of the MGF-110-9L gene is sufficient to reduce the virulence of the ASFV [41]. It has also been reported that the protein encoded by the MGF505-7R gene promotes virulence and pathogenesis by inhibiting JAK-mediated signaling [42]. In addition, it has been shown that MGF360/505 gene products can participate in the regulation of the immune response of infected cells. Thus, deletion of MGF360-11L or MGF505-11R genes led to an increase in the production of type I interferon [43,44]. Further study of the function of ASFV genes, as well as the study of genetic changes in comparison with biological properties, could help in the development of approaches to the design of vaccine strains.

Other changes in the genome of attenuated strains may also be important for understanding the pathogenesis of ASF infection. We found multiple insertions into the central hypervariable region (CVR) of the B602L gene of the attenuated strain. These inserts of tandem repeats led to an elongation of the protein encoded by the B602L gene by 36 amino acids. The data obtained are in good agreement with previously detected variations of the B602L gene. For example, studies of genetic changes in two naturally attenuated isolates of the ASFV have shown that the B602L gene of the NH/P68 virus is 234 bp (78aa) longer than that of its virulent homologue L60, and the B602L gene of the OURT 88/3 virus is 12 bp (4aa) longer than that of NH/P68 [45]. It is worth noting that the amino acid sequence does not have a frameshift as a result of insertions, and the remaining regions of the B602L protein, with the exception of CVR, demonstrate a high degree of identity. It remains unclear how the length of the variable region of the B602L gene affects the properties of the encoded protein, but it cannot be excluded that these changes may affect the replication of the virus or its virulence. It is also possible that insertions and deletions in intergenic regions may affect the expression of virus genes, which may be important for its weakening.

Attenuation of ASFV strains obtained by sequential passages in cell lines is important not only for vaccine research but is also a useful tool for studying the relationship of genetic changes with pathogenesis. Achieving a balance between reducing virulence and maintaining immunogenicity is a key task in the development of vaccine candidate strains.

## 5. Conclusions

African swine fever (ASF) is an acute hemorrhagic disease of pigs caused by the virus (ASFV). The use of attenuated strains of the ASFV in the development of the vaccine made it possible to protect animals from infection with a homologous virulent virus. However, not much is known about the genetic determinants of ASFV attenuation. The attenuated Congo-a (KK262) virus demonstrated differences in in vivo replication and virulence compared to its virulent homologue Congo-v (K49), but it did not lose the ability to grow in vitro in the primary culture of pig macrophages. Complete genome sequencing of the attenuated KK262 strain revealed an 8.8 kb deletion in the left variable region of the genome compared to K49. In addition, insertions in the B602L gene, genetic changes in intergenic regions and missense mutations in eight genes were detected. We hope that our data will contribute to a better understanding of ASFV attenuation and identification of potential virulence genes for further development of effective vaccines.

## Figures and Tables

**Table 1:** Differences in genomes of KK262 strain compared to K49 strain.

Gene/Protein Region	Position in ASFV K49 Genome (bp)	Nucleotide/Amino Acid Change in ASFV KK262 Genome
L60L/MGF_360-3L (IGR)	5619	A-C
MGF_110-10L/110-11L	12,362	Frameshift insertion
ASFVK49_0660/0665(IGR)	15,684–15,696	Deletion
ASFVK49_0700	17,587	Insertion in Tandem Repeat
ASFVK49_0700/0720 (IGR)	17,896–17,900	Deletion
MGF_360-9L/10Lb (IGR)	26,107	Insertion in Tandem Repeat
MGF_360-10La- MGF_505-3R	26,013–34,824	Deletion
MGF505-10R	45,679	A-G
ACD_00600/A104R (IGR)	46,964	Insertion in Tandem Repeat
F165R (putative protein pF165R)	59,592	Substitution Arg-Gly
EP402R (CD2v)	73,629	C-T
	74,130	T-C
M1249L (minor capsid protein pM1249L)	76,023	C-T
C315R (putative protein pC315R)	87,043	Substitution Arg-His
C315R/C147L (IGR)	87,849	Insertion in Tandem Repeat
C962L (helicase-like protein pC962R)	89,657	T-C
B602L	101,523	Insertion in Tandem Repeat
	101,559	Insertion in Tandem Repeat
	101,615	Insertion in Tandem Repeat
G1211R (DNA-directed DNA polymerase)	113,487	T-G
CP530R (polyprotein pp62)	126,126	Substitution Leu-Val
NP1450L (RNA polymerase subunit 1)	131,323	Substitution Ser-Pro
D205R (mRNA decapping protein)	137,251	Substitution Ile-Thr
	137,812	Substitution Met-Val
	137,871	Substitution Glu-Lys
E199L (membrane fusion protein pE199L)	165,767	Substitution Phe-Ile
E120R (structural protein p14.5)	167,788	Substitution Asp-Gly
	167,799	Substitution Glu-Lys
I8L (non-essential early protein)	181,127	Substitution Cys-Arg

## Data Availability

The complete genome sequences of the Congo-a (KK262) strain were deposited in GenBank with accession number OM249788.1.

## Supplementary Materials

The following supporting information can be downloaded at https://www.mdpi.com/article/10.3390/v15061373/s1: Figure S1: Body temperature (A), clinical signs (B) in pigs inoculated with ASFV strains Congo-a (KK262) and Congo-v (K49); Figure S2: Antibody response to ASFV detected in pigs IM inoculated with 10^6^ HAD50 ASFV Congo-a or 10^3^ HAD50 ASFV Congo-v; Figure S3: Kinetics of ASFV genomes in blood (A) and viral load in organs (B) detected in pigs IM inoculated with 10^6^ HAD50 ASFV Congo-a (#1/1-1/10) or 10^3^ HAD50 ASFV Congo-v (#2/1-2/10).
