# Supplementary material for: Comparison of Attenuated and Virulent Strains of African Swine Fever Virus Genotype I and Serogroup 2
Source: Viruses. 2023 Jun 14;15(6):1373. doi: 10.3390/v15061373 (PMC10301422; doi:10.3390/v15061373)
Supplement: Supplementary file 1 [file viruses-15-01373-s001.zip › Supplementary Materials.pdf]

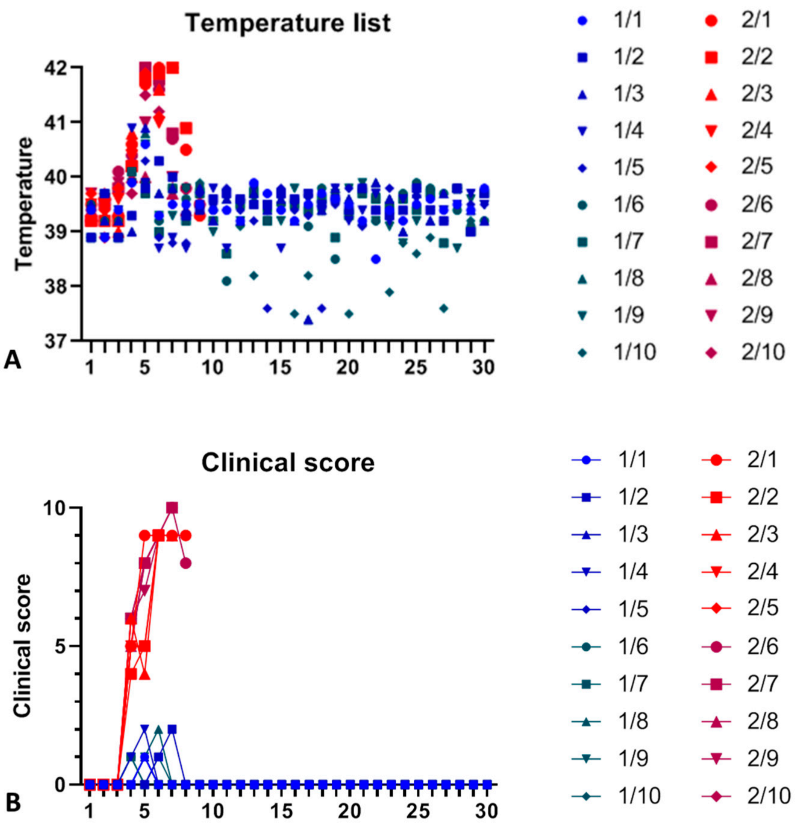

Figure S1. Body temperature (A), clinical signs (B) in pigs inoculated with ASFV strains Congo-a (KK262) and Congo-v (K49). Pigs were inoculated with Congo-a (strain KK-262) (#1/1-1/10) or Congo-v (strain K49) (#2/1-2/10). Data on body temperature and clinical score are given for individual animals from each group. The analysis was conducted using Graphpad Prism software version 8.0.1.

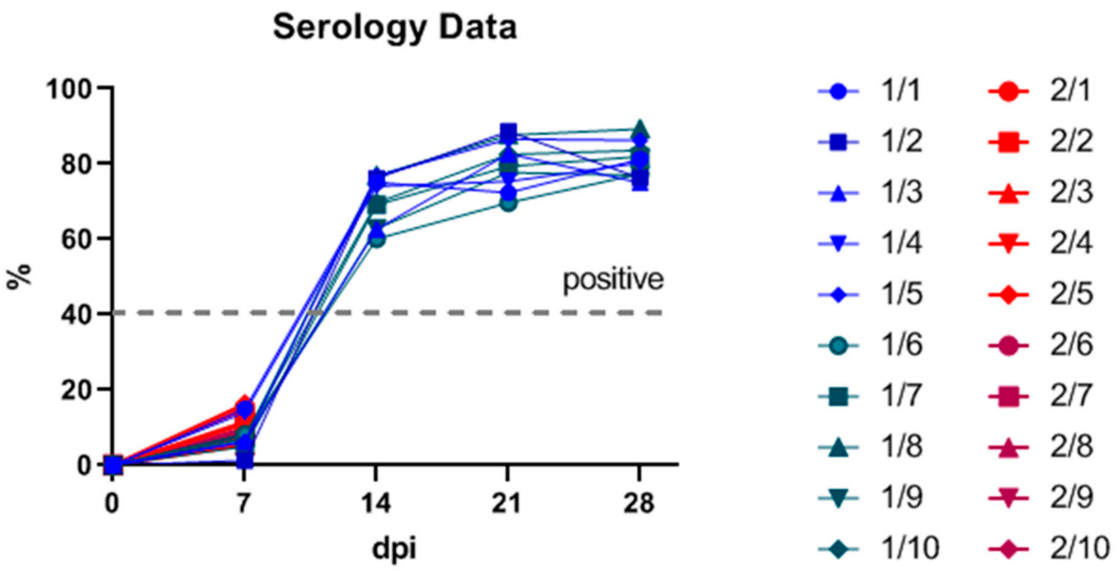

Figure S2. Antibody response to ASFV detected in pigs IM inoculated with 10^6 HAD50 ASFV Congo-a or 10^3 HAD50 ASFV Congo-v. Antibody responses to ASFV measured using ELISA in pigs immunized with ASFV Congo-a (#1/1-1/10) or Congo-v (#2/1-2/10) at 0–28 dpi. The dashed line indicates a threshold value of 40%. Data on antibody response to ASFV are given for individual animals from each group. The analysis was conducted using Graphpad Prism software version 8.0.1.

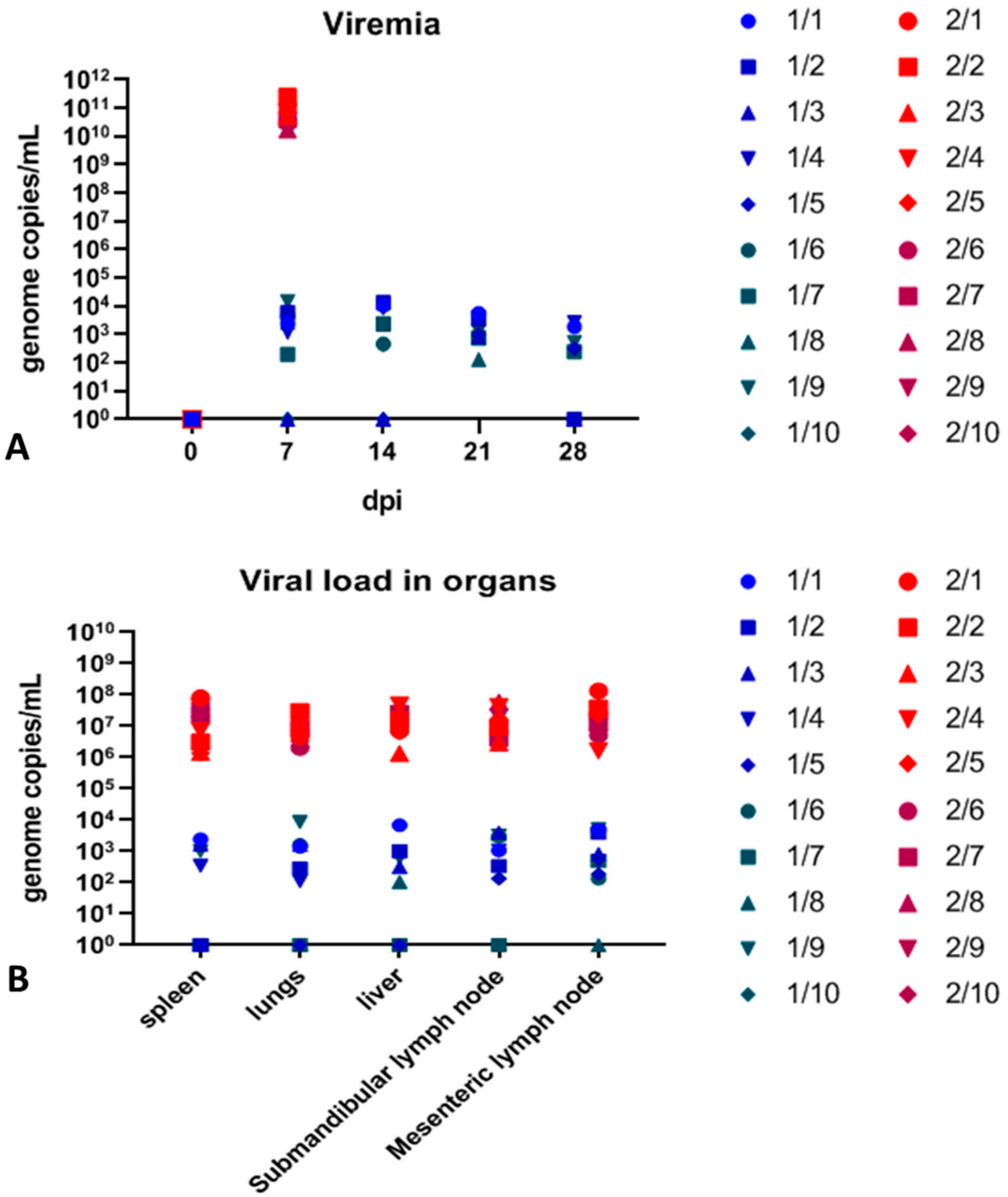

Figure S3. Kinetics of ASFV genomes in blood (A) and viral load in organs (B) detected in pigs IM inoculated with 10^6 HAD50 ASFV Congo-a (#1/1-1/10) or 10^3 HAD50 ASFV Congo-v (#2/1-2/10). The detection of the ASFV genome was conducted in blood (A) and organs (B) by the qPCR. The results are presented as genome copies/mL. Data on the amount of ASFV genome in blood and organ samples are given for individual animals from each group. The analysis was conducted using Graphpad Prism software version 8.0.1.
